# The effect of the SARS-CoV-2 pandemic in the compliance of hand hygiene in ICU in a tertiary hospital

**DOI:** 10.1093/eurpub/ckac131.381

**Published:** 2022-10-25

**Authors:** M Russo Sanjuanbenito, MJ Urquijo Valdovinos, AB Sánchez de la Ventana, M Ibáñez Rebe, I Carnicer Gil, T Blanco Maestro, L Mascaraque Sánchez, M Fuentes Ferrer

**Affiliations:** 1 Preventive Medicine Department, Hospital Clinico San Carlos, Madrid, Spain; 2 Intensive Care Unit, Hospital Clinico San Carlos, Madrid, Spain; 3 Nephrology Department, Hospital Clinico San Carlos, Madrid, Spain; 4 Cardiology Department, Hospital Clinico San Carlos, Madrid, Spain; 5 Traumatology Department, Hospital Clinico San Carlos, Madrid, Spain

## Abstract

**Background:**

Hand hygiene (HH) is one of the main preventive methods for healthcare-associated infections. Our aim is to compare the level of HH compliance in 2021, SARS-CoV-2 pandemic period, with the previous 2019 period.

**Methods:**

Descriptive cross-sectional study of direct observation on compliance of HH in adult intensive care unit (AICU) and neonatal intensive care unit (NICU) in a third-level hospital in Madrid during the years 2019 and 2021. Trained healthcare workers observed and recorded hand hygiene opportunities (HHO) among staff (nurse, nurses’ aide, physicians and orderly) using the World Health Organization's “My Five Moments for Hand Hygiene” tool. All observations of professional that attended patients in SARS-CoV-2 isolation units were excluded.

**Results:**

Overall, there was a total of 1199 HHO, 961 in AICU (2019:466; 2021:495) and 238 in NICU (2019:122; 2021:116). HH compliance in AICU improved from 57.5% in 2019 to 65.9% in 2021 (p = 0.008) and remained unchanged in NICU (2019:90.2% vs 2021:88.8%; p = 0.730). In AICU all professional categories, except nurse’s aide, improved HH compliance being statistically significant in physicians (2019:33.8% vs 2021:50.0%; p = 0.009). In relation to the 5 moments, an increase in HH compliance was observed in: before and after touching a patient/after touching patient surroundings, being only statistically significant in moment after touching a patient (2019:69.4% vs 2021:83.7%; p = 0.001). In NICU there were no significant changes between the two periods regarding to the professional category or the five moments of HH. In NICU there were no significant changes between the two periods regarding to the professional category or the five moments of HH.

**Conclusions:**

The increase in compliance of HH within the adult ICUs demonstrates this adaptation, stating a behavioural change in the habits of professionals. This increase was no observed in the NICU since their compliance was already extremely high.

**Key messages:**

• There has been an increase in HH compliance in the AICU.

• The HH method has varied, becoming COVID-19 specific.

